# Engineering Multifunctional Surfaces: Unveiling the Extraordinary Electrical, Thermal, and Magnetic Properties of CVD-Synthesized Carbon Nanotubes via Nanofabrication

**DOI:** 10.1063/4.0000872

**Published:** 2025-10-27

**Authors:** Salah OUDJERTLI Habilitation to Direct Research and Senior Researcher Class- A -

## Abstract

The remarkable properties of carbon nanotubes (CNTs) have spurred extensive research into their development and application as multifunctional materials for advanced technologies. This study explores the synthesis of CNTs via chemical vapor deposition (CVD) and nanofabrication techniques, aiming to achieve precise control over their morphology and enhance their material quality. Comprehensive characterization is conducted to evaluate their electronic, thermal, and magnetic properties, emphasizing their intrinsic anisotropy and the quantum mechanical phenomena that underpin their exceptional performance. Electrical measurements demonstrate ultra-high carrier mobility, while thermal conductivity analysis confirms their superior heat dissipation capabilities. Furthermore, investigations into their magnetic behavior reveal promising functionalities for potential spintronic applications. By deepening the understanding of CNT properties, this work contributes to optimizing their performance for energy storage, sensing, and next- generation electronic devices, fostering their integration into cutting-edge technological advancements.

